# Supplementary material for: CRISPR tiling deletion screens reveal functional enhancers and allelic compensation effects (ACE) on SIN3A transcription
Source: Nat Commun. 2026 Mar 25;17:4396. doi: 10.1038/s41467-026-70933-y (PMC13181091; doi:10.1038/s41467-026-70933-y)
Supplement: Supplementary file 1 — Supplementary Information [file 41467_2026_70933_MOESM1_ESM.pdf]

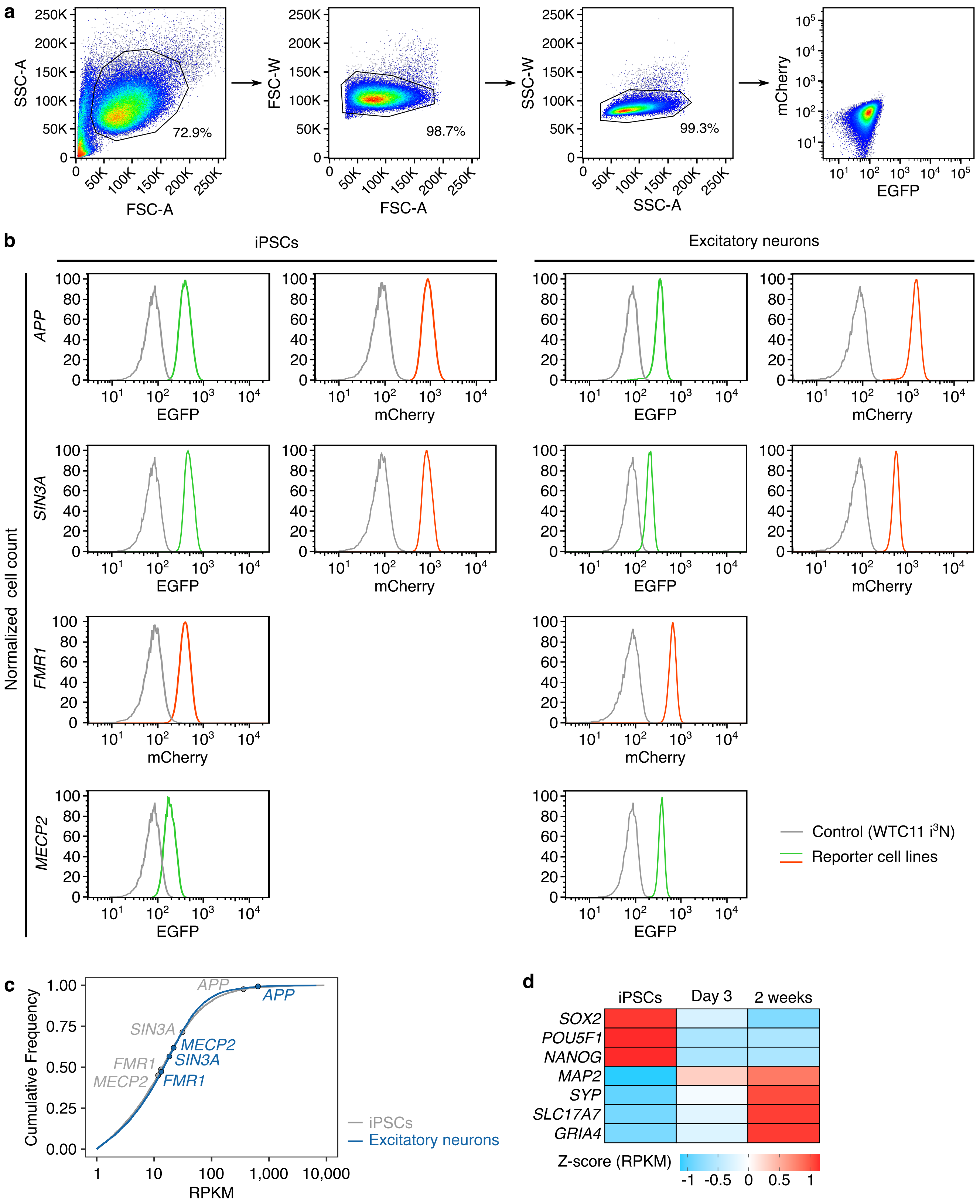

Supplementary Figure 1. **Engineered reporter cell lines and gene expression. a,** Representative plots showing the gating strategy used for flow cytometry analysis and FACS. First, cells were separated from debris based on forward scatter area (FSC-A) and side scatter area (SSC-A). Then, single cells were separated using single cell gates based on the area and width metrics of forward scatter (FSC-A vs. FSC-W) and side scatter (SSC-A vs. SSC-W). Further, the gates for EGFP and mCherry signal baselines were set using cells without EGFP and mCherry signals. **b,** Flow cytometry plots showing the expression of EGFP and mCherry reporters in APP-EGFP/mCherry, SIN3A-EGFP/mCherry, FMR1-mCherry, and MECP2-EGFP reporter cell lines. The expression of reporters was checked in both iPSCs and excitatory neurons. Gray lines are signals from negative control cells, WTC11 i^{3}N. **c,** RNA-seq data shows the expression of *APP*, *FMR1, MECP2,* and *SIN3A* in iPSCs and 2-week excitatory neurons. The genes were ranked on RPKM. **d,** The expression of cell type marker genes in iPSCs and excitatory neurons.

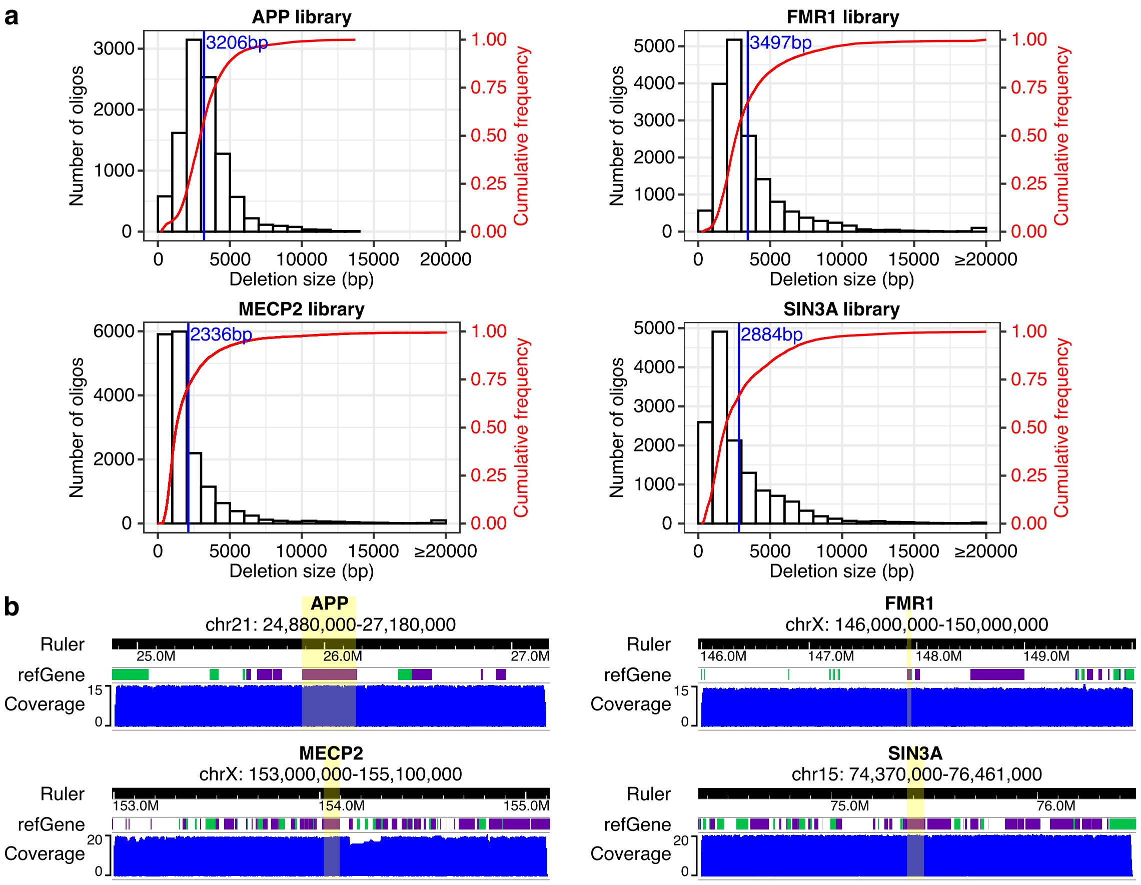

c

pgRNAs	APP	FMR1	MECP2	SIN3A
EGFP targeting	5	0	5	5
mCherry targeting	5	5	0	5
Non-targeting control	467	234	130	446
Targeting	10,311	16,466	17,158	14,015
Total	10,788	16,705	17,293	14,471

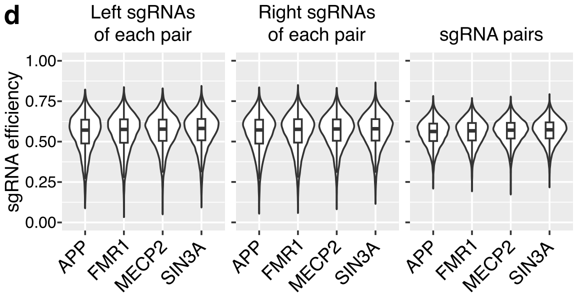

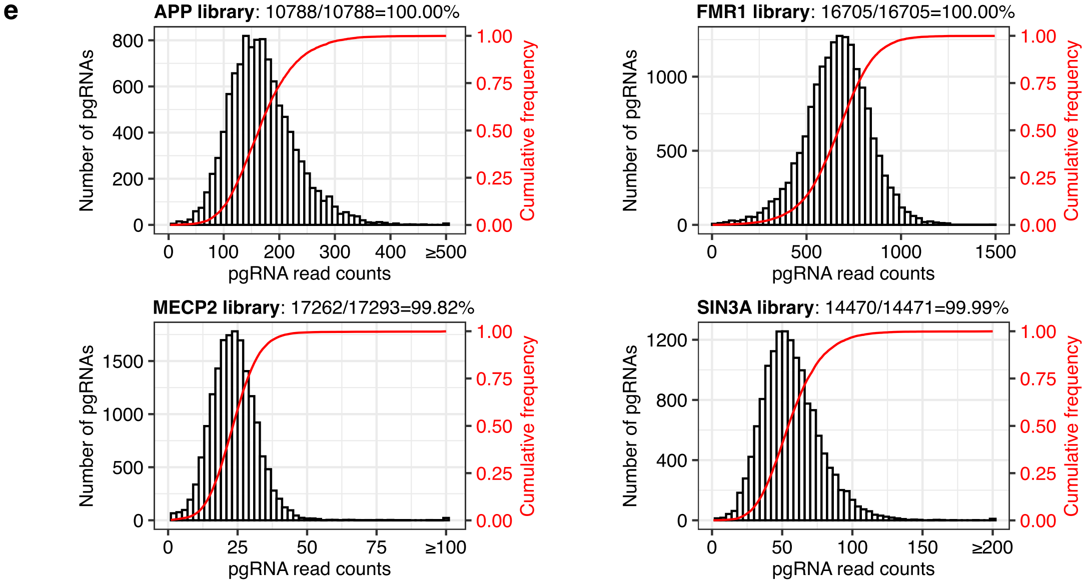

Supplementary Figure 2. **pgRNA libraries of *APP*, *FMR1*, *MECP2*, and *SIN3A*. a,** The distribution of deletion size of pgRNA libraries. Blue lines indicate the average deletion size of each pgRNA library. **b,** The coverage of pgRNA libraries. The gene body regions of each gene were labeled with yellow. **c,** The composition of pgRNA libraries. **d,** The sgRNA efficiency (Rule Set 2 score) of sgRNAs in each pgRNA library. Violin plots show the distributions of Rule Set 2 score within each group, and boxplots indicate the median, interquartile range (IQR), Q1 − 1.5 × IQR and Q3 + 1.5 × IQR. **e,** The distribution of pgRNA read counts and cumulative frequency in cloned plasmid libraries. More than 99% of designed pgRNA were recovered in each plasmid library.

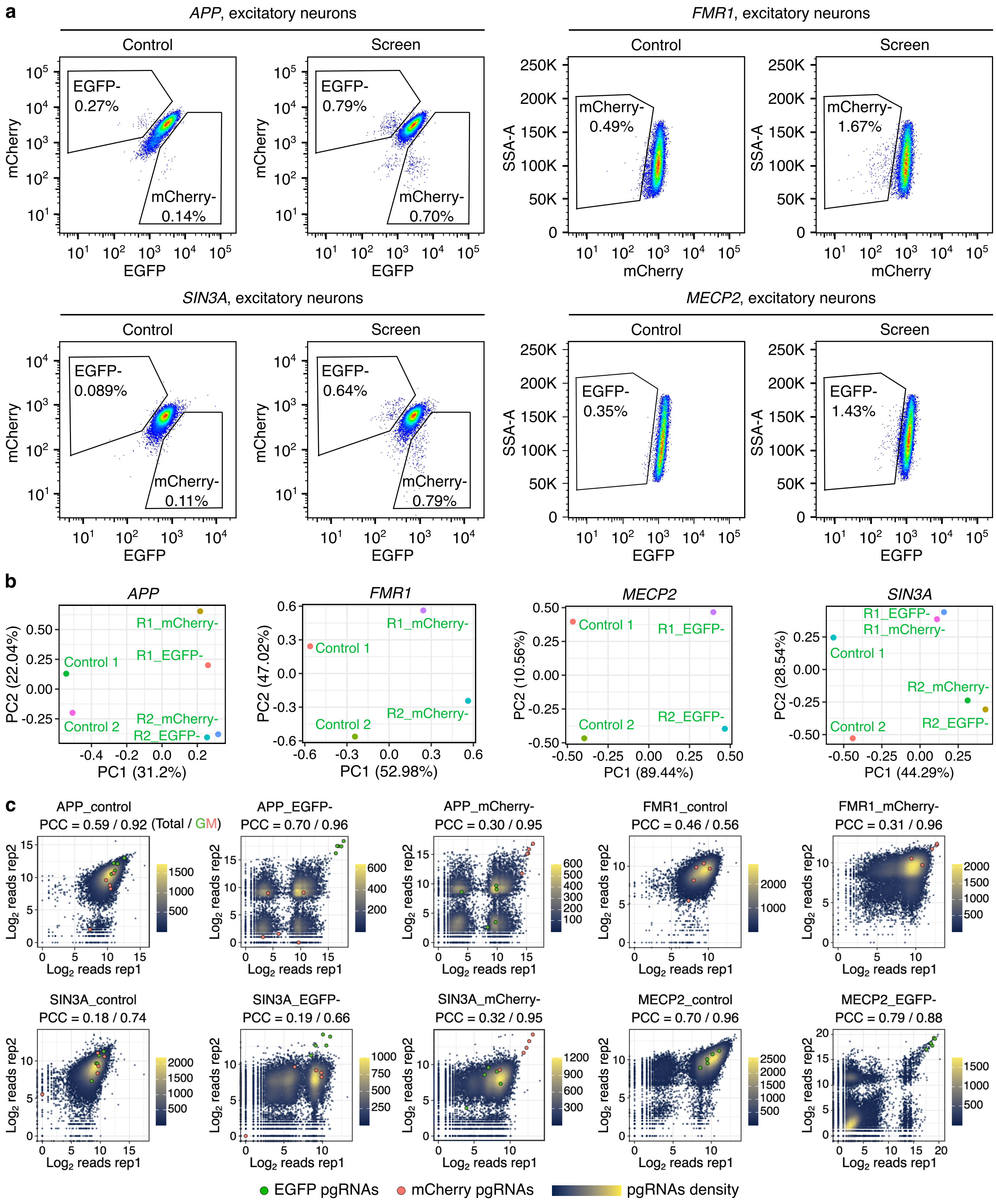

Supplementary Figure 3. CREST-seq screens. a, Representative FACS plots showing the sorting strategies used in the CREST-seq screens. Reporter cells without pgRNA library infection were used as the control for each screen. **b,** PCA analysis of CREST-seq screens. **c,** Scatter plots showing the distribution of individual pgRNAs in each screen. The Pearson correlation coefficient (PCC) values for all pgRNAs (Total) and positive control pgRNAs (EGFP and mCherry pgRNAs: GM) are indicated in each plot.

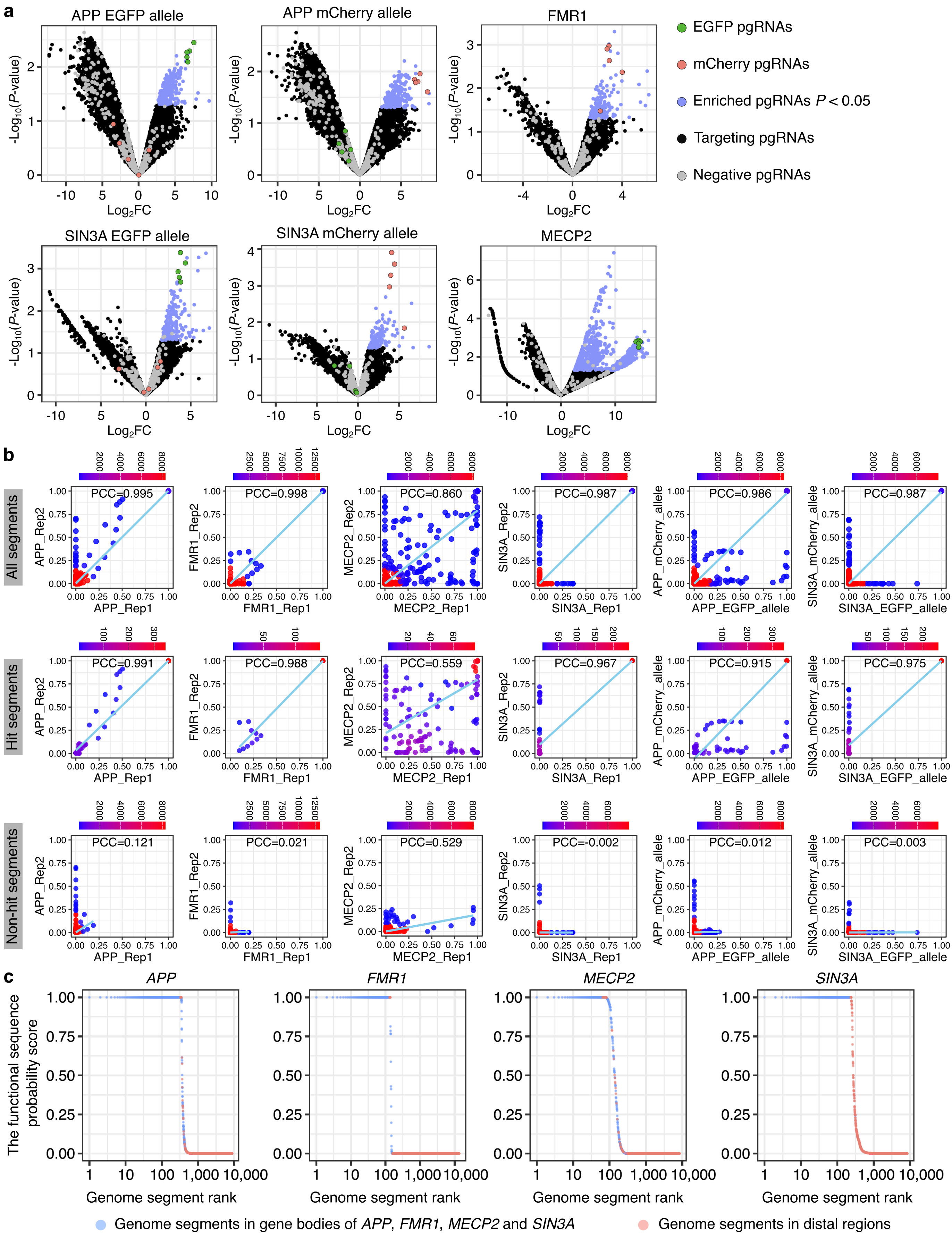

Supplementary Figure 4. Quality control and data analysis of CREST-seq screens. a, Volcano plots showing the fold changes and *P* values for each screen. *APP* and *SIN3A* screening data from EGFP and mCherry allele are shown separately. **b,** Scatterplots of RELICS probability scores. The scores are shown for all segments, hit segments, and non-hit segments. The Pearson correlation coefficient (PCC) values and linear regression lines are indicated in each plot. **c,** Functional sequence probability scores of genome segments from RELICS analysis for each screen.

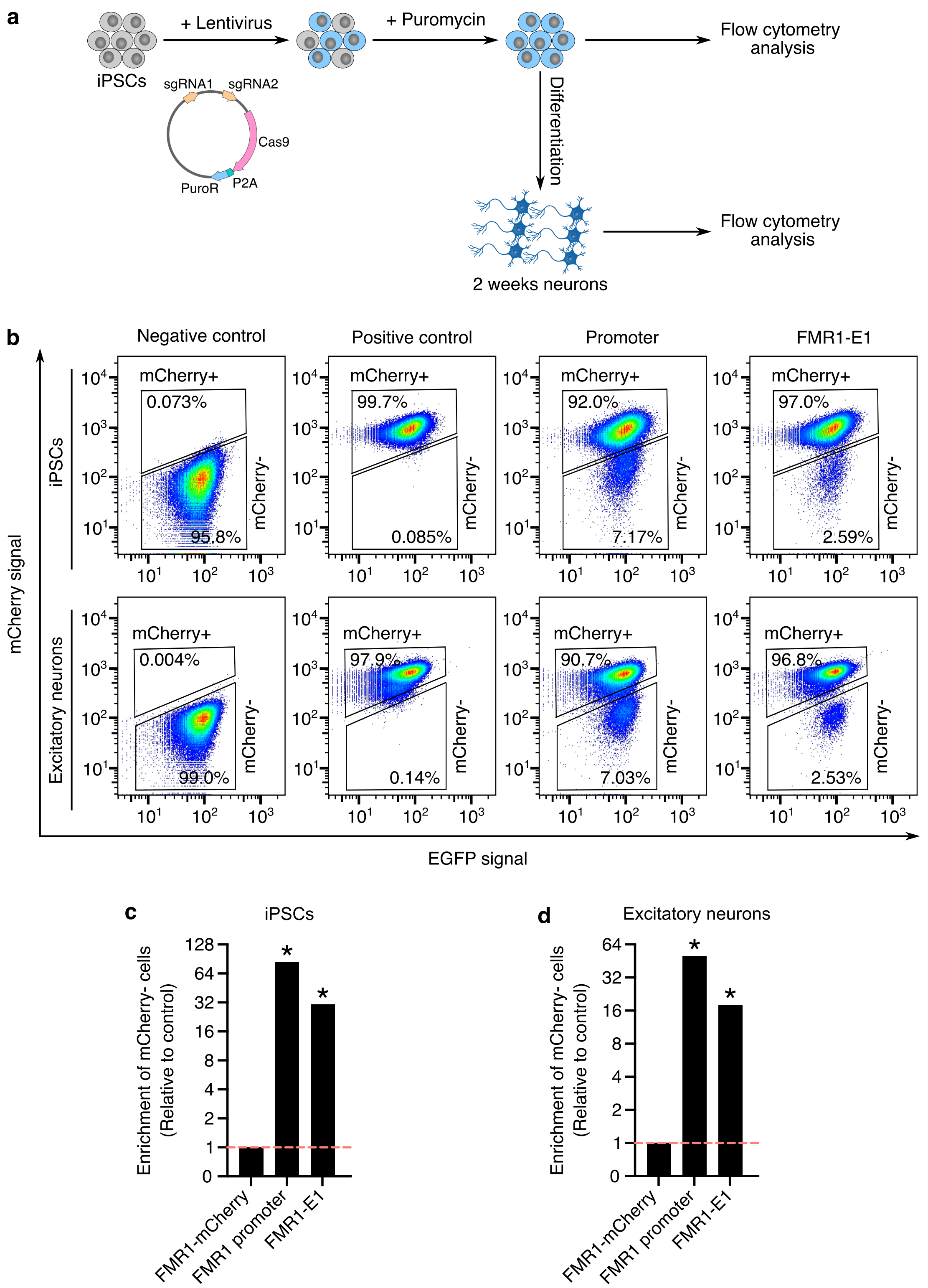

Supplementary Figure 5. Enhancer validation strategy and validation of *FMR1* enhancer. a, The flow cytometry based strategy for enhancer validation. **b,** Flow cytometry plots showing the percentage of cells with reduced FMR1-mCherry expression in each condition. The negative control is the WTC11 i^{3}N cells. The positive control is the FMR1-mCherry reporter cells. **c,** Bar graphs showing the significance of the relative enrichment of cells with reduced expression of FMR1-mCherry compared to positive control cells. *P* values were determined using the two-sided Fisher's exact test. * $P < 0.0001$. Source data are provided as a Source Data file.

a

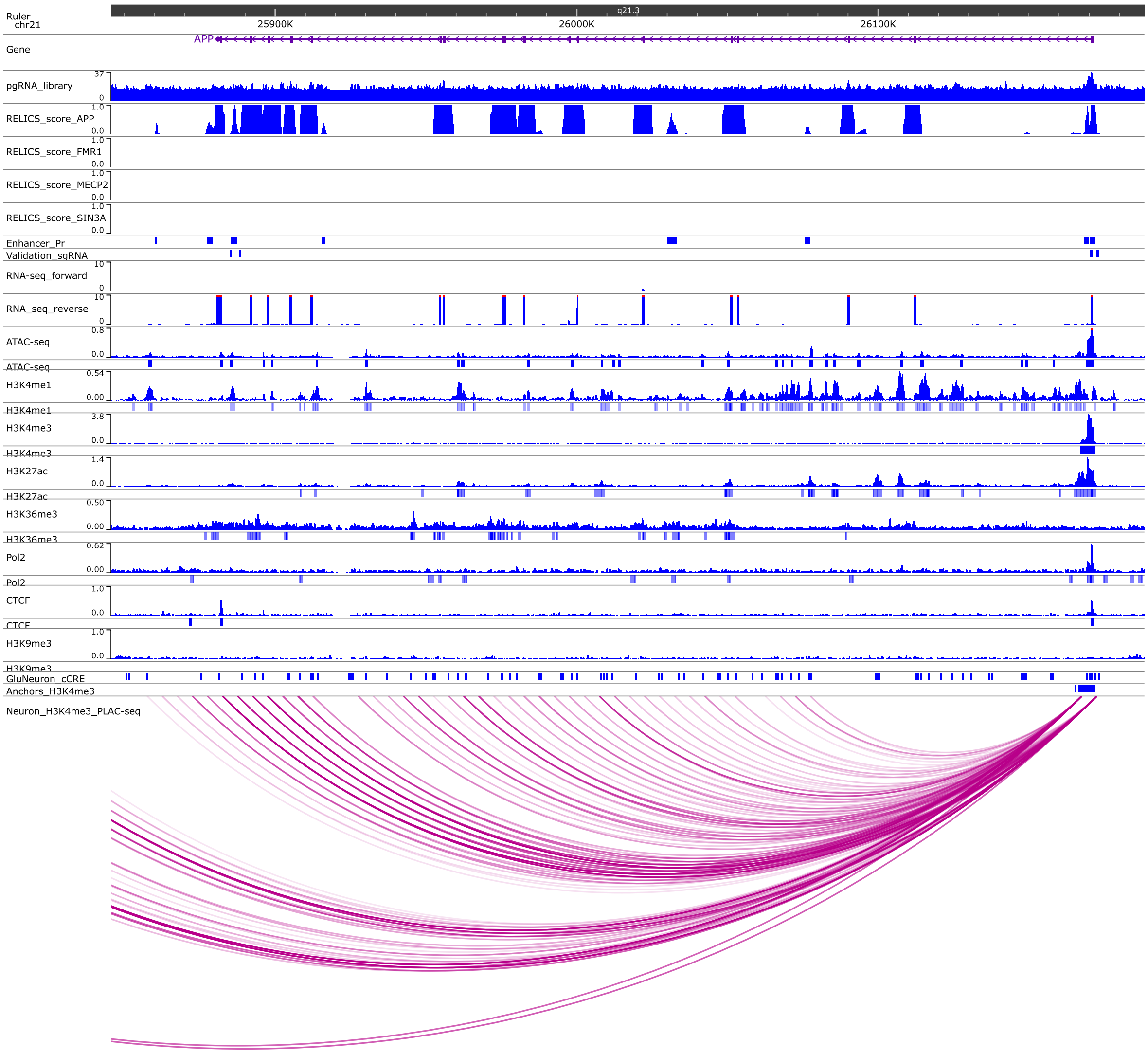
Ruler
chr21
q21.3
25900K
26000K
26100K
Gene
APP
pgRNA_library
RELICS_score_APP
RELICS_score_FMR1
RELICS_score_MECP2
RELICS_score_SIN3A
Enhancer_Pr
Validation_sgRNA
RNA-seq_forward
RNA_seq_reverse
ATAC-seq
H3K4me1
H3K4me3
H3K27ac
H3K36me3
Pol2
CTCF
H3K9me3
GluNeuron_cCRE
Anchors_H3K4me3
Neuron_H3K4me3_PLAC-seq

b

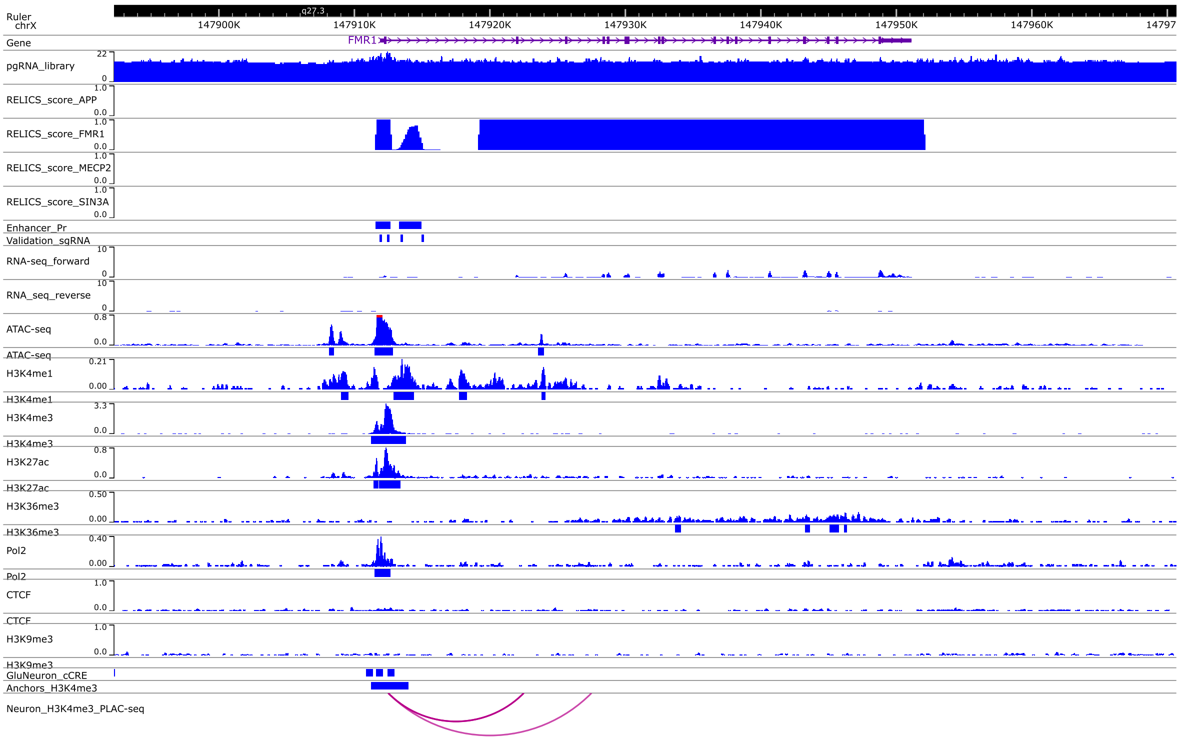
Ruler
chrX
q27.3
147900K
147910K
147920K
147930K
147940K
147950K
147960K
14797
Gene
FMR1
pgRNA_library
RELICS_score_APP
RELICS_score_FMR1
RELICS_score_MECP2
RELICS_score_SIN3A
Enhancer_Pr
Validation_sgRNA
RNA-seq_forward
RNA_seq_reverse
ATAC-seq
H3K4me1
H3K4me3
H3K27ac
H3K36me3
Pol2
CTCF
H3K9me3
GluNeuron_cCRE
Anchors_H3K4me3
Neuron_H3K4me3_PLAC-seq

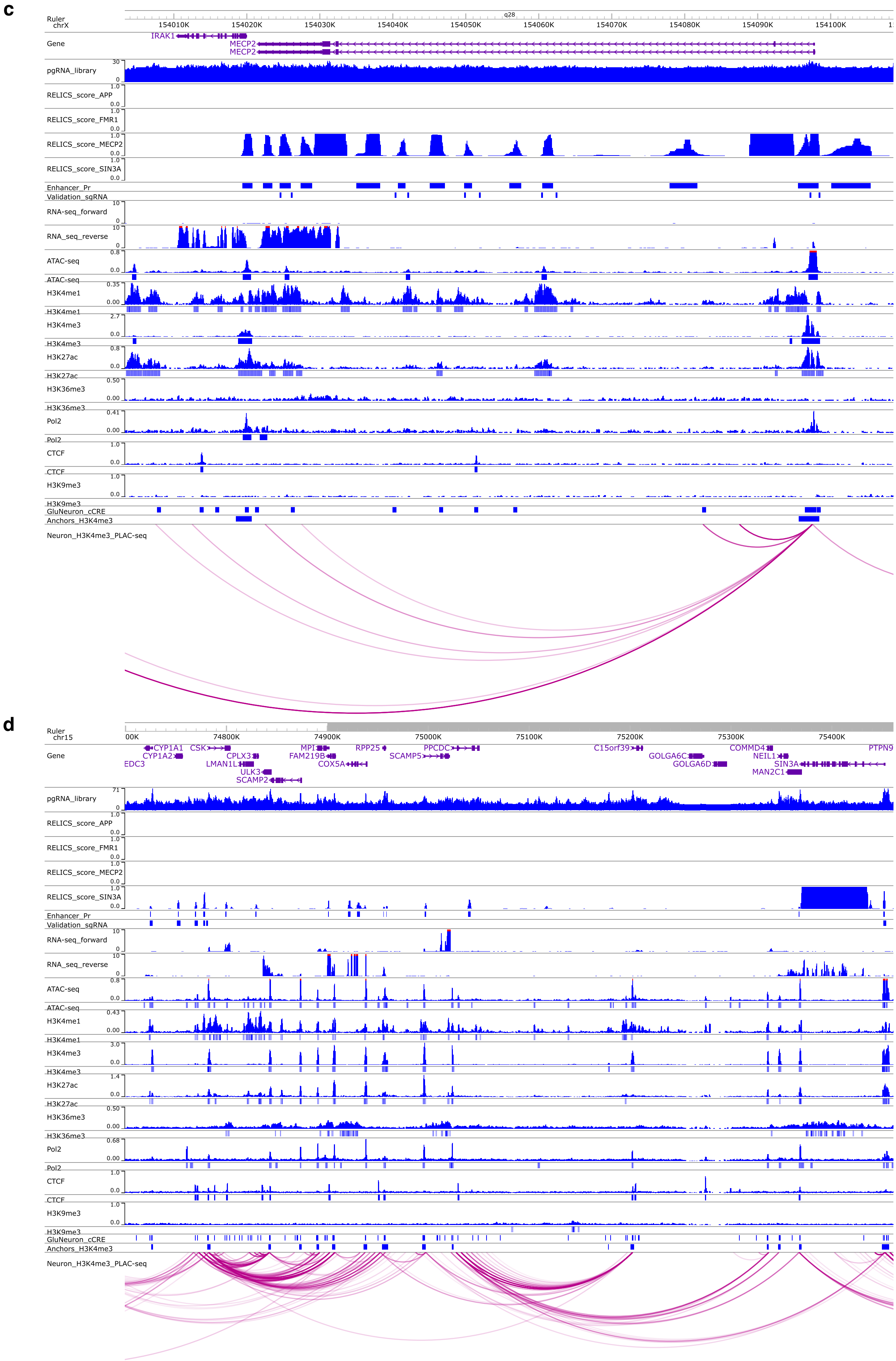

Supplementary Figure 6. Genome browser snapshots of the loci containing enhancers in validation experiments. a-d, Tracks at each locus containing pgRNA library, RELICS score, identified enhancers, sgRNAs used for validation experiments, RNA-seq data, chromatin markers, and H3K4me3 mediated PLAC-seq.

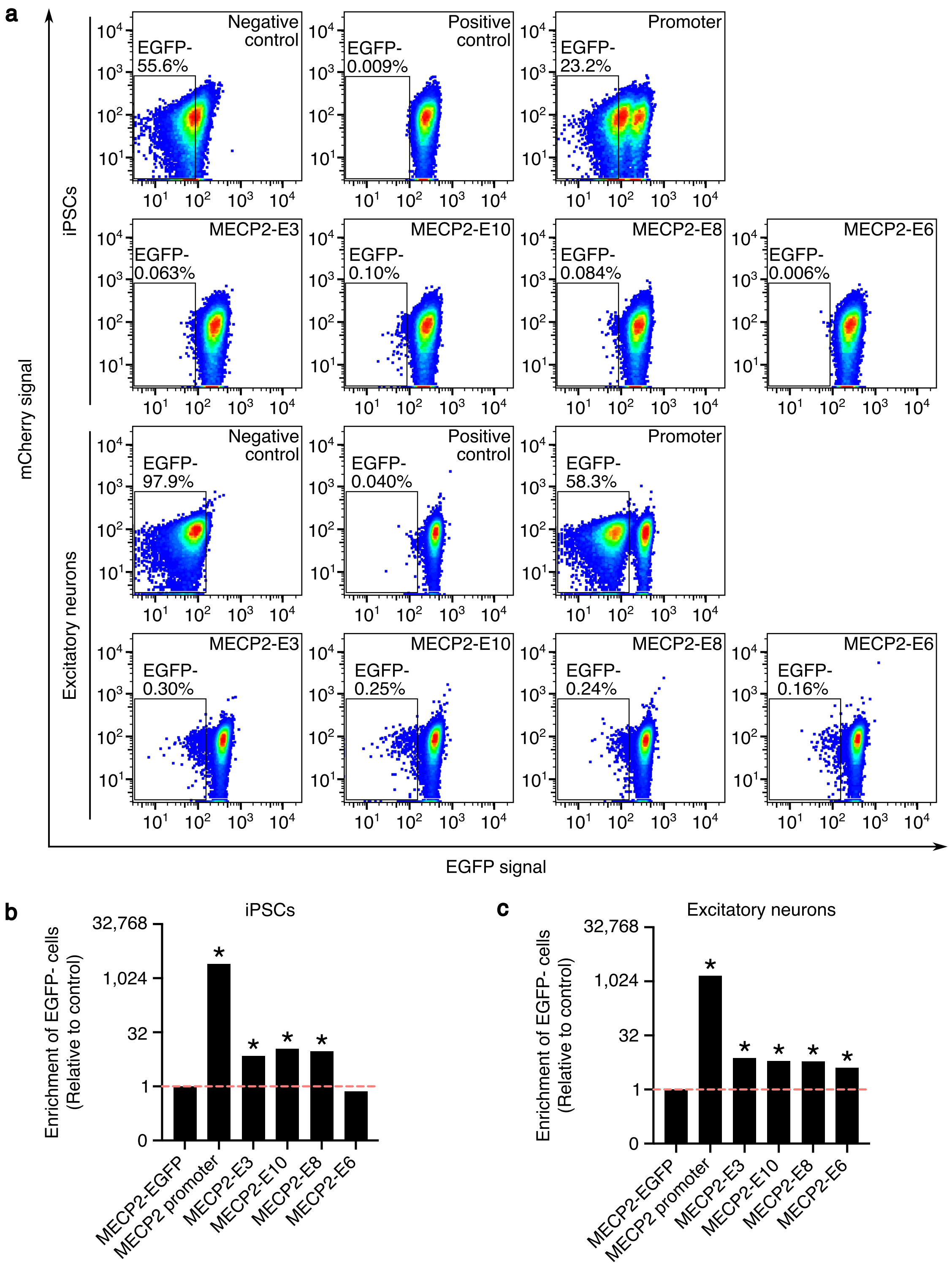

Supplementary Figure 7. *MECP2* enhancer validations. a, Flow cytometry plots showing the percentage of cells with reduced MECP2-EGFP expression in each condition. The negative control is the WTC11 i^{3}N cells. The positive control is the MECP2-EGFP reporter cells. **b,c,** Bar graphs showing the significance of the relative enrichment of cells with reduced expression of MECP2-EGFP compared to positive control cells. *P* values were determined using the two-sided Fisher's exact test. * $P < 0.0001$. Source data are provided as a Source Data file.

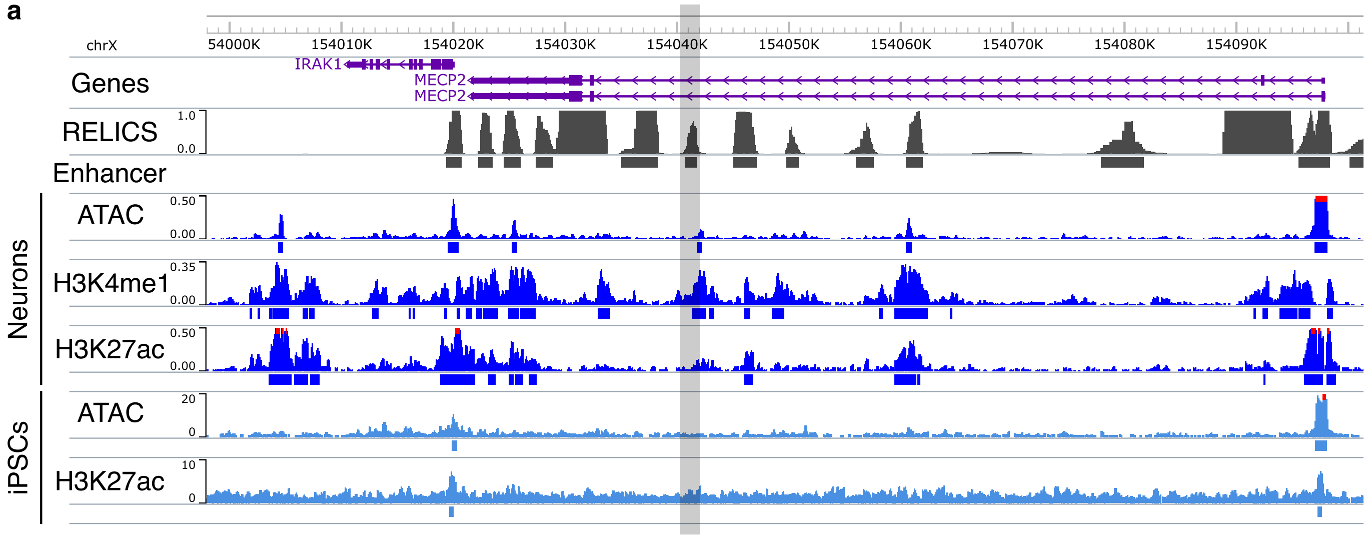

Supplementary Figure 8. **Genome browser snapshot of the *MECP2* locus. a,** The gray region represents the MECP2-E6 deletion region in the validation experiment.

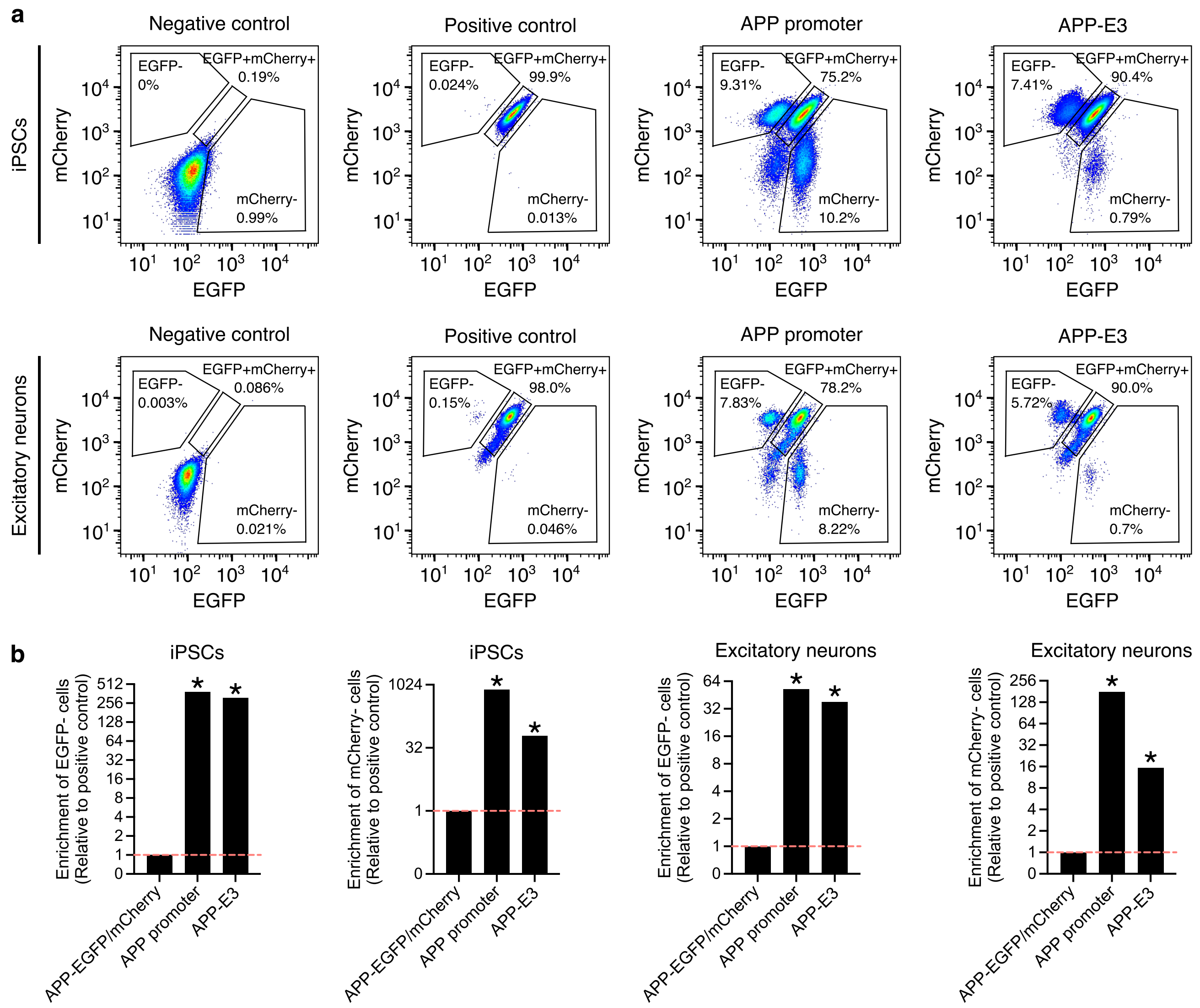

Supplementary Figure 9. ***APP*** **enhancer validation. a,** Flow cytometry plots showing the percentage of cells with reduced expression of APP-EGFP or APP-mCherry signals in each condition. The negative control is the WTC11 i³N cells. The positive control is the APP-EGFP/mCherry reporter cells. **b,** Bar graphs showing the significance of the relative enrichment of cells with reduced expression of APP-EGFP or APP-mCherry compared to positive control cells. *P* values were determined using the two-sided Fisher's exact test. * $P < 0.0001$. Source data are provided as a Source Data file.

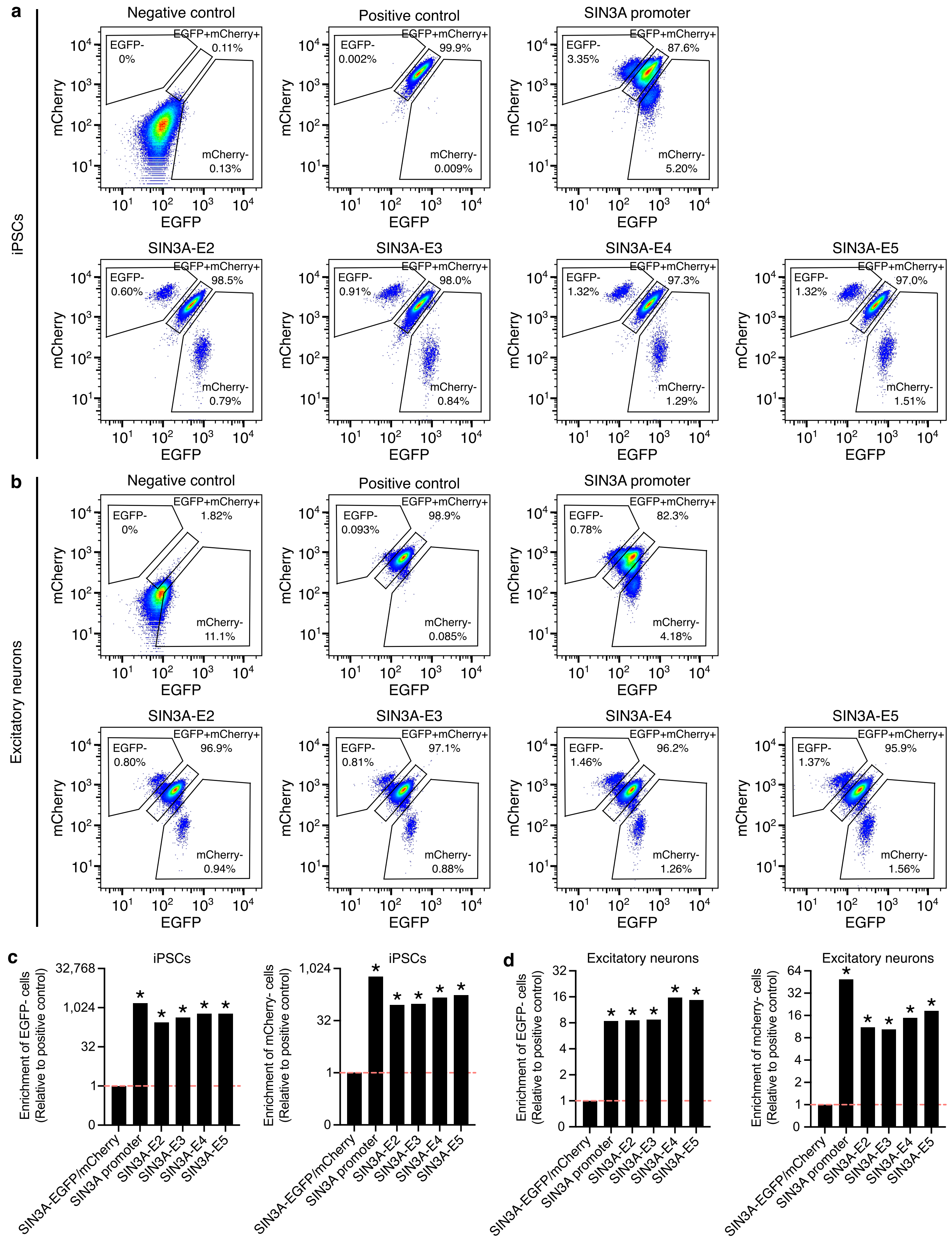

Supplementary Figure 10. *SIN3A* enhancer validations. a,b, Flow cytometry plots showing the percentage of cells with reduced expression of SIN3A-EGFP or SIN3A-mCherry in each condition. The negative control is the WTC11 i^{3}N cells. The positive control is the SIN3A-EGFP/mCherry reporter cells. **c,d,** Bar graphs showing the significance of the relative enrichment of cells with reduced expression of SIN3A-EGFP or SIN3A-mCherry compared to positive control cells. *P* values were determined using the two-sided Fisher's exact test. * $P < 0.0001$. Source data are provided as a Source Data file.

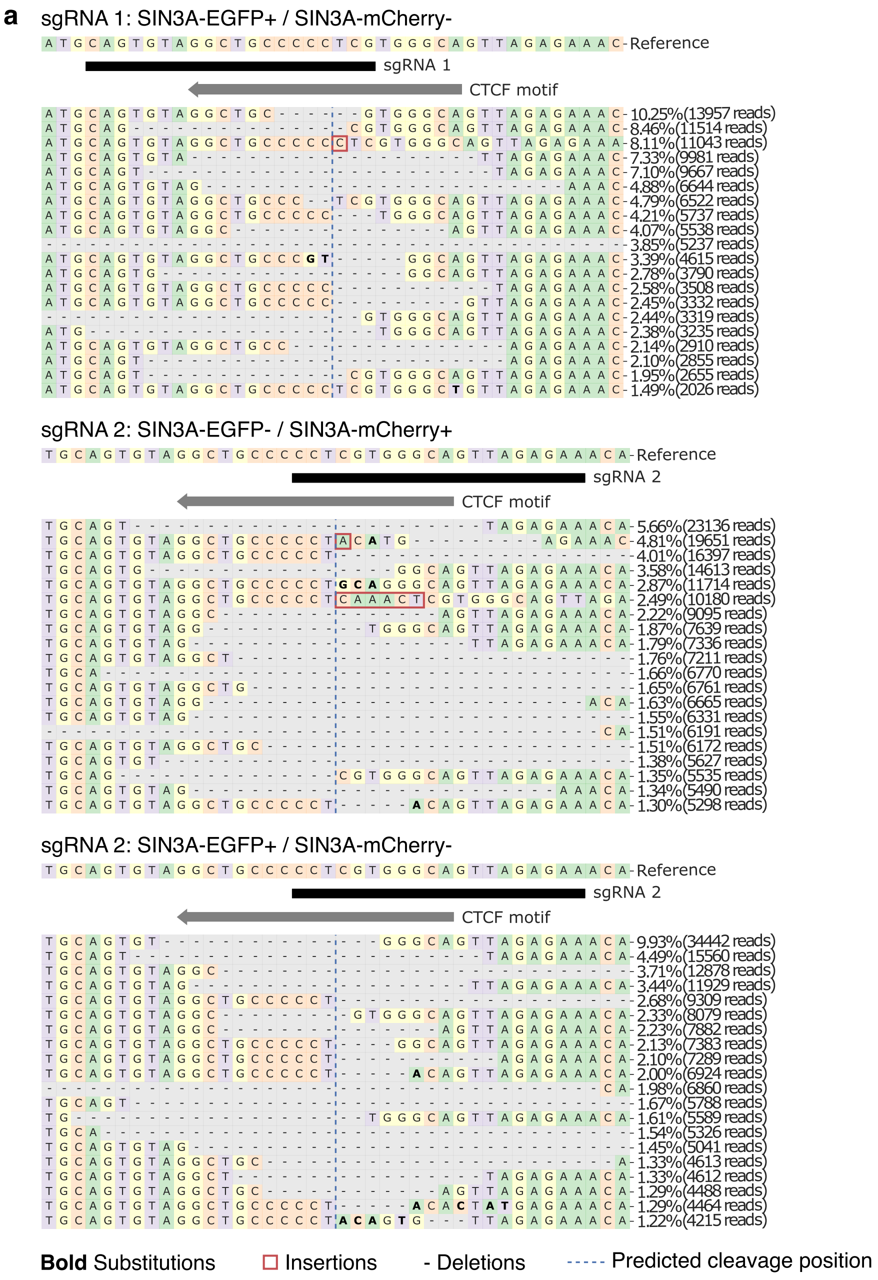

Supplementary Figure 11. Editing outcomes of CTCF sgRNAs. a, CRISPResso2 analysis of the targeted sequencing data shows the genome editing outcomes at the CTCF motif in the cells with reduced expression of SIN3A-EGFP or SIN3A-mCherry.

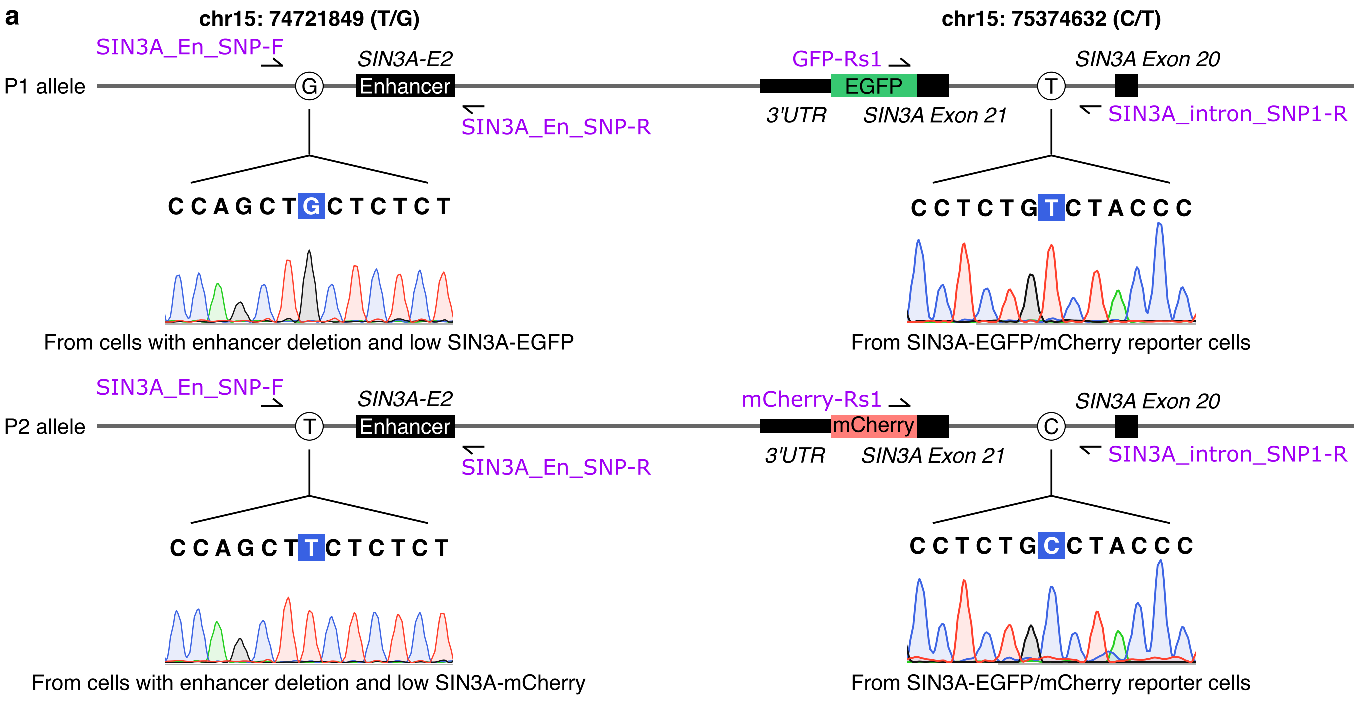

Supplementary Figure 12. *cis*-regulation of *SIN3A* by the SIN3A-E2 enhancer. a, Sanger sequencing data showing the genotype of each allele of *SIN3A* enhancer and *SIN3A*. P1 and P2 alleles are identified using the phased variants in WTC11 genome. Both *SIN3A* enhancer region and *SIN3A* region are amplified using genomic DNA from indicated cells, and the phased variants in amplified regions are confirmed using Sanger sequencing.

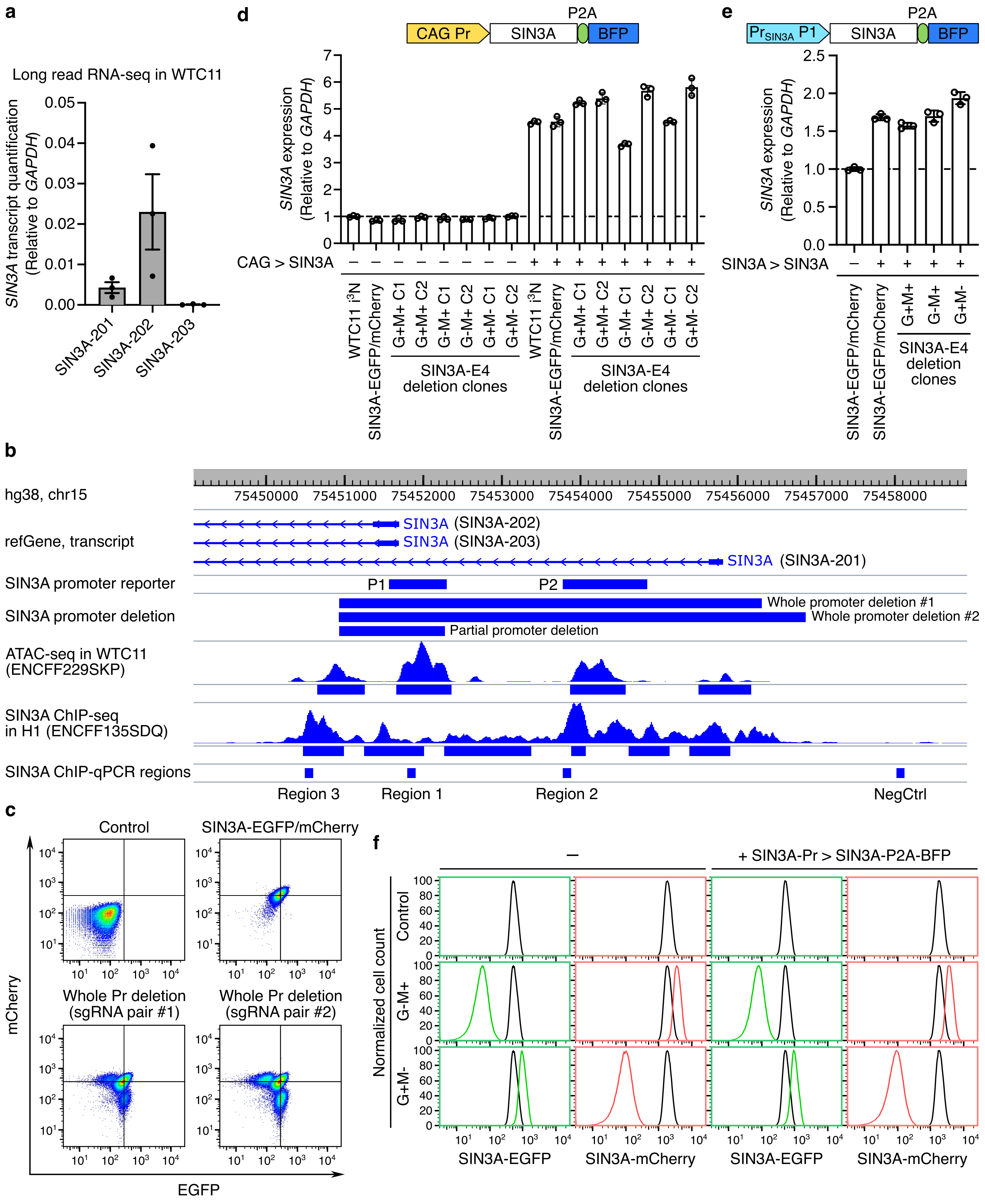

Supplementary Figure 13. *SIN3A* ectopic expression and *SIN3A* promoter related assays. a, The expression of *SIN3A* transcripts from long read RNA-seq data in WTC11 cells. Data are mean ± SEM from three biological replicates. **b,** WashU Epigenome Browser snapshot showing *SIN3A* transcripts from refGene, *SIN3A* promoter deletion region in validation experiments, two promoter regions used for *SIN3A* promoter reporter assay, ATAC-seq signal in WTC11 iPSCs, and *SIN3A* ChIP-seq signals in H1 cells, and regions checked with ChIP-qPCR. **c,** Flow cytometry plots showing the EGFP and mCherry signals in control cells (WTC11 i^{3}N), SIN3A-EGFP/mCherry reporter cells, and SIN3A promoter deletion cells. **d,e,** RT-qPCR results showing the expression levels of *SIN3A* in control conditions and overexpression conditions. Data are mean ± SD from three technical replicates. **f,** Flow cytometry plots showing the SIN3A-EGFP and SIN3A-mCherry signals in control cells and SIN3A-E4 deletion clones with and without *SIN3A* promoter controlled ectopic *SIN3A* expression. SIN3A-EGFP/mCherry reporter cells were used as control. Source data are provided as a Source Data file.

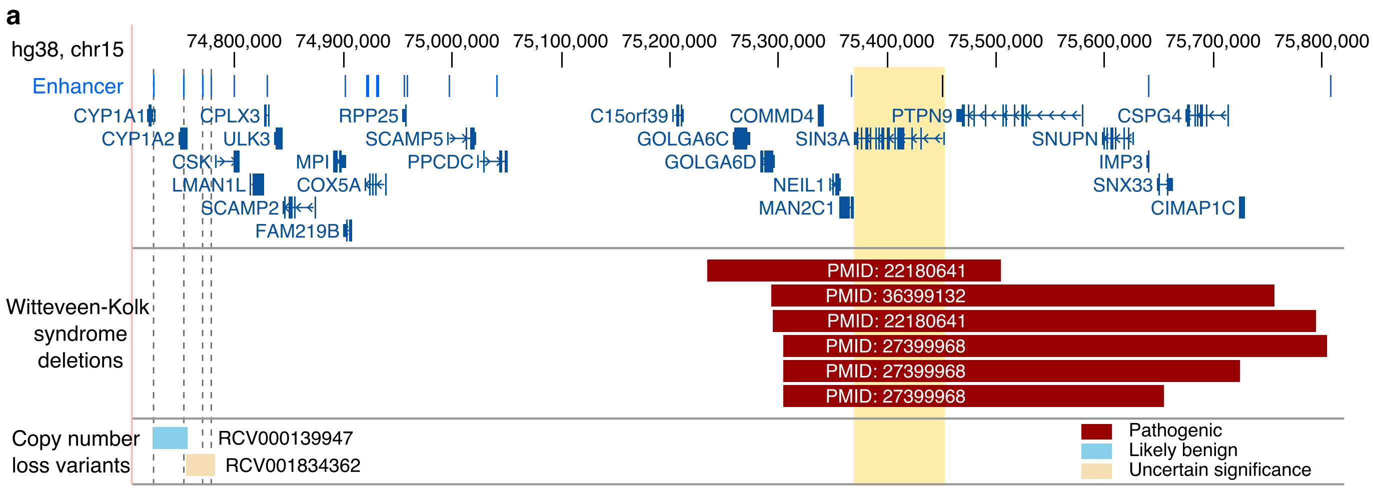

Supplementary Figure 14. Overlap between *SIN3A* enhancers, the *SIN3A* gene, and genetic variants. a, Genetic variants include heterozygous deletions from Witteveen-Kolk syndrome patients and two copy number loss variants reported in ClinVar.

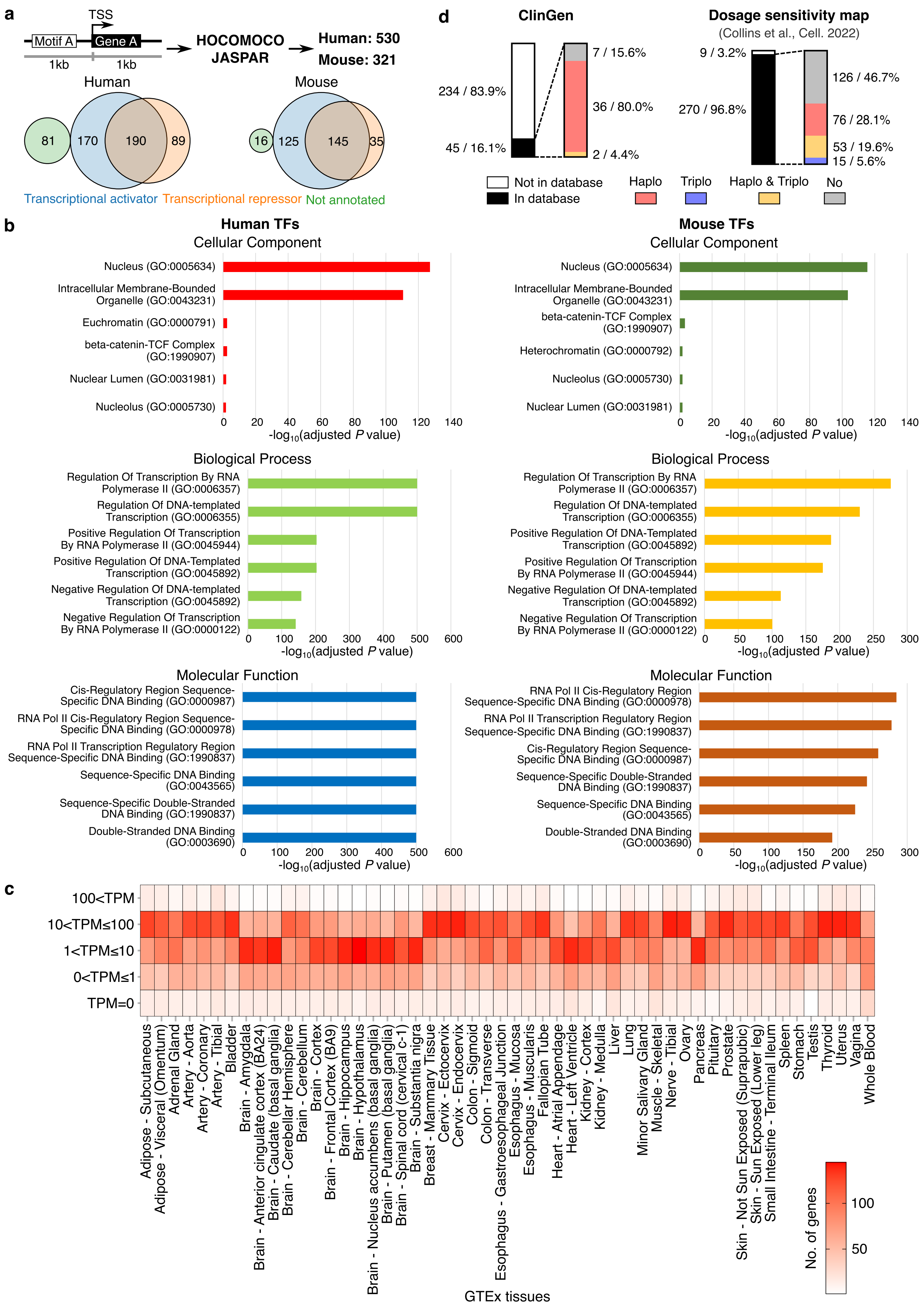
a
TSS
Motif A
Gene A
1kb
1kb
HOCOMOCO
JASPAR
Human: 530
Mouse: 321
Human
81
170
190
89
Mouse
16
125
145
35
Transcriptional activator
Transcriptional repressor
Not annotated
d
ClinGen
234 / 83.9%
45 / 16.1%
7 / 15.6%
36 / 80.0%
2 / 4.4%
Dosage sensitivity map
(Collins et al., Cell. 2022)
9 / 3.2%
270 / 96.8%
126 / 46.7%
76 / 28.1%
53 / 19.6%
15 / 5.6%
Not in database
In database
Haplo
Triplo
Haplo & Triplo
No
b
Human TFs
Cellular Component
Nucleus (GO:0005634)
Intracellular Membrane-Bounded Organelle (GO:0043231)
Euchromatin (GO:0000791)
beta-catenin-TCF Complex (GO:1990907)
Nuclear Lumen (GO:0031981)
Nucleolus (GO:0005730)
0 20 40 60 80 100 120 140
-log10(adjusted P value)
Mouse TFs
Cellular Component
Nucleus (GO:0005634)
Intracellular Membrane-Bounded Organelle (GO:0043231)
beta-catenin-TCF Complex (GO:1990907)
Heterochromatin (GO:0000792)
Nucleolus (GO:0005730)
Nuclear Lumen (GO:0031981)
0 20 40 60 80 100 120 140
-log10(adjusted P value)
Biological Process
Regulation Of Transcription By RNA Polymerase II (GO:0006357)
Regulation Of DNA-templated Transcription (GO:0006355)
Positive Regulation Of Transcription By RNA Polymerase II (GO:0045944)
Positive Regulation Of DNA-Templated Transcription (GO:0045892)
Negative Regulation Of DNA-templated Transcription (GO:0045892)
Negative Regulation Of Transcription By RNA Polymerase II (GO:0000122)
0 100 200 300 400 500 600
-log10(adjusted P value)
Biological Process
Regulation Of Transcription By RNA Polymerase II (GO:0006357)
Regulation Of DNA-templated Transcription (GO:0006355)
Positive Regulation Of DNA-Templated Transcription (GO:0045892)
Positive Regulation Of Transcription By RNA Polymerase II (GO:0045944)
Negative Regulation Of DNA-templated Transcription (GO:0045892)
Negative Regulation Of Transcription By RNA Polymerase II (GO:0000122)
0 50 100 150 200 250 300
-log10(adjusted P value)
Molecular Function
Cis-Regulatory Region Sequence-Specific DNA Binding (GO:0000987)
RNA Pol II Cis-Regulatory Region Sequence-Specific DNA Binding (GO:0000978)
RNA Pol II Transcription Regulatory Region Sequence-Specific DNA Binding (GO:1990837)
Sequence-Specific DNA Binding (GO:0043565)
Sequence-Specific Double-Stranded DNA Binding (GO:1990837)
Double-Stranded DNA Binding (GO:0003690)
0 100 200 300 400 500 600
-log10(adjusted P value)
Molecular Function
RNA Pol II Cis-Regulatory Region Sequence-Specific DNA Binding (GO:0000978)
RNA Pol II Transcription Regulatory Region Sequence-Specific DNA Binding (GO:1990837)
Cis-Regulatory Region Sequence-Specific DNA Binding (GO:0000987)
Sequence-Specific Double-Stranded DNA Binding (GO:1990837)
Sequence-Specific DNA Binding (GO:0043565)
Double-Stranded DNA Binding (GO:0003690)
0 50 100 150 200 250 300
-log10(adjusted P value)
c
100<TPM
10<TPM≤100
1<TPM≤10
0<TPM≤1
TPM=0
Adipose - Subcutaneous
Adipose - Visceral (Omentum)
Adrenal Gland
Artery - Aorta
Artery - Coronary
Artery - Tibial
Bladder
Brain - Amygdala
Brain - Anterior cingulate cortex (BA24)
Brain - Caudate (basal ganglia)
Brain - Cerebellar Hemisphere
Brain - Cerebellum
Brain - Cortex
Brain - Frontal Cortex (BA9)
Brain - Hippocampus
Brain - Hypothalamus
Brain - Nucleus accumbens (basal ganglia)
Brain - Putamen (basal ganglia)
Brain - Spinal cord (cervical c-1)
Brain - Substantia nigra
Breast - Mammary Tissue
Cervix - Ectocervix
Cervix - Endocervix
Colon - Sigmoid
Colon - Transverse
Esophagus - Gastroesophageal Junction
Esophagus - Mucosa
Esophagus - Muscularis
Fallopian Tube
Heart - Atrial Appendage
Heart - Left Ventricle
Kidney - Cortex
Kidney - Medulla
Liver
Lung
Minor Salivary Gland
Muscle - Skeletal
Nerve - Tibial
Ovary
Pancreas
Pituitary
Prostate
Skin - Not Sun Exposed (Suprapubic)
Skin - Sun Exposed (Lower leg)
Small Intestine - Terminal Ileum
Spleen
Stomach
Testis
Thyroid
Uterus
Vagina
Whole Blood
GTEx tissues
No. of genes
100
50

Supplementary Figure 15. Transcriptional compensation is associated with gene dosage sensitivity. a, The strategy used for identifying candidate genes with transcriptional compensation. Venn diagrams show the distribution of transcriptional activators and transcriptional repressors in 530 human transcription factors (TFs) and 321 mouse TFs. **b,** The significant enrichment of human and mouse TFs in cellular component, biological process, and molecular function. **c,** The expression of the identified candidate transcriptional compensation genes (transcriptional repressor) in human tissues. The expression data were obtained from GTEx. **d,** The distribution of identified candidate transcriptional compensation genes in ClinGen and Dosage sensitivity map. Haplo: haploinsufficiency. Triplo: triplosensitivity.

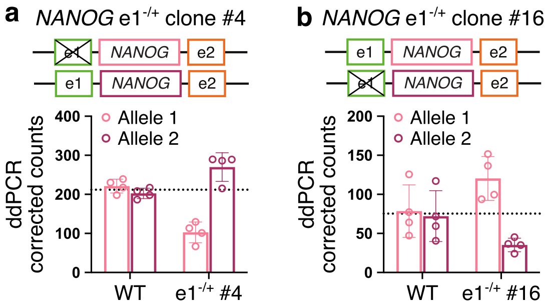

Supplementary Figure 16. Allelic expression of *NANOG* in *NANOG* e1 enhancer deletion clones. a,b, ddPCR results of *NANOG* allelic expression in *NANOG* e1 enhancer deletion clones. Data were reanalyzed from Yan *et al.* (PMID: 40446796). Source data are provided as a Source Data file.
