# Supplementary material for: CRISPR tiling deletion screens reveal functional enhancers and allelic compensation effects (ACE) on SIN3A transcription
Source: Nat Commun. 2026 Mar 25;17:4396. doi: 10.1038/s41467-026-70933-y (PMC13181091; doi:10.1038/s41467-026-70933-y)
Supplement: Supplementary file 2 — Description of Additional Supplementary Files [file 41467_2026_70933_MOESM2_ESM.pdf]

**Description of Additional Supplementary Files**

**Supplementary Data 1.** List of identified enhancers

**Supplementary Data 2.** DNA oligo sequences for donor cloning, RT-qPCR, genotyping and library preparation

**Supplementary Data 3.** sgRNA sequences for enhancer validation and generating reporter cell lines

**Supplementary Data 4.** shRNA sequences for SIN3A knockdown

**Supplementary Data 5.** Information of datasets used in this study

**Supplementary Data 6.** Candidate transcriptional compensation genes
