# Supplementary material for: CRISPR tiling deletion screens reveal functional enhancers and allelic compensation effects (ACE) on SIN3A transcription
Source: Nat Commun. 2026 Mar 25;17:4396. doi: 10.1038/s41467-026-70933-y (PMC13181091; doi:10.1038/s41467-026-70933-y)
Supplement: Supplementary file 4 — Reporting Summary [file 41467_2026_70933_MOESM4_ESM.pdf]

nature portfolio

Corresponding author(s): Yin Shen

Last updated by author(s): Jan 1, 2026

Reporting Summary

Nature Portfolio wishes to improve the reproducibility of the work that we publish. This form provides structure for consistency and transparency in reporting. For further information on Nature Portfolio policies, see our Editorial Policies and the Editorial Policy Checklist.

Statistics

For all statistical analyses, confirm that the following items are present in the figure legend, table legend, main text, or Methods section.

n/a	Confirmed	
☐	☒	The exact sample size (*n*) for each experimental group/condition, given as a discrete number and unit of measurement
☐	☒	A statement on whether measurements were taken from distinct samples or whether the same sample was measured repeatedly
☐	☒	The statistical test(s) used AND whether they are one- or two-sided<br>*Only common tests should be described solely by name; describe more complex techniques in the Methods section.*
☒	☐	A description of all covariates tested
☒	☐	A description of any assumptions or corrections, such as tests of normality and adjustment for multiple comparisons
☐	☒	A full description of the statistical parameters including central tendency (e.g. means) or other basic estimates (e.g. regression coefficient) AND variation (e.g. standard deviation) or associated estimates of uncertainty (e.g. confidence intervals)
☐	☒	For null hypothesis testing, the test statistic (e.g. *F*, *t*, *r*) with confidence intervals, effect sizes, degrees of freedom and *P* value noted<br>*Give P values as exact values whenever suitable.*
☒	☐	For Bayesian analysis, information on the choice of priors and Markov chain Monte Carlo settings
☒	☐	For hierarchical and complex designs, identification of the appropriate level for tests and full reporting of outcomes
☐	☒	Estimates of effect sizes (e.g. Cohen's *d*, Pearson's *r*), indicating how they were calculated

Our web collection on statistics for biologists contains articles on many of the points above.

Software and code

Policy information about availability of computer code

Data collection	Sequencing data was obtained from the Illumina NovaSeq 6000 platform.
Data analysis	We used the following software: BWA (bwa-0.7.17), RELICS (v.2.0), bedtools (v2.26.0), chip-seq-pipeline2 (v2.1.6), FlowJo (v10.7.2), FIMO (v5.5.4), Enrichr.

For manuscripts utilizing custom algorithms or software that are central to the research but not yet described in published literature, software must be made available to editors and reviewers. We strongly encourage code deposition in a community repository (e.g. GitHub). See the Nature Portfolio guidelines for submitting code & software for further information.

Data

Policy information about availability of data

All manuscripts must include a data availability statement. This statement should provide the following information, where applicable:

- Accession codes, unique identifiers, or web links for publicly available datasets
- A description of any restrictions on data availability
- For clinical datasets or third party data, please ensure that the statement adheres to our policy

The CRISPR screen datasets used in this study are available at the ENCODE portal (www.encodeproject.org) and accession numbers are ENCSR783CGW (APP pgRNA plasmid library), ENCSR364KFC (APP control), ENCSR678GDA (Low APP-EGFP), ENCSR952RDF (Low APP-mCherry), ENCSR493NRD (SIN3A pgRNA plasmid library), ENCSR284PQK (SIN3A control), ENCSR113CEG (Low SIN3A-mCherry), ENCSR750UIY (Low SIN3A-EGFP), ENCSR888FDQ (FMR1 pgRNA plasmid library), ENCSR466IBU

(FMR1 control), ENCSR562YXE (Low FMR1-mCherry), ENCSR473BRJ (MECP2 pgRNA plasmid library), ENCSR072YHQ (MECP2 control), and ENCSR119JRG (Low MECP2-EGFP). Public datasets used in this study are listed in Supplementary Table 5. Data can be visualized on the WashU Epigenome Browser using the following session: https://epigenomegateway.wustl.edu/browser2022/?genome=hg38&sessionFile=https://shen-xren.s3.us-west-1.amazonaws.com/CREST-seq_NC/eg-session-YzGAc9x4n-ce92ef10-72ad-11f0-b5d2-a94bbd38dced.json. Tracks include pgRNA library, RNA-seq, ATAC-seq, chromatin marks, cCREs annotated in excitatory neurons from the human brain samples, H3K4me3 mediated PLAC-seq, RELICS scores, identified functional enhancers for each screen, and sgRNAs used for validation experiments. Source data are provided with this paper.

Research involving human participants, their data, or biological material

Policy information about studies with human participants or human data. See also policy information about sex, gender (identity/presentation), and sexual orientation and race, ethnicity and racism.

Reporting on sex and gender	N/A
Reporting on race, ethnicity, or other socially relevant groupings	N/A
Population characteristics	N/A
Recruitment	N/A
Ethics oversight	N/A

Note that full information on the approval of the study protocol must also be provided in the manuscript.

Field-specific reporting

Please select the one below that is the best fit for your research. If you are not sure, read the appropriate sections before making your selection.

☒ Life sciences ☐ Behavioural & social sciences ☐ Ecological, evolutionary & environmental sciences

For a reference copy of the document with all sections, see nature.com/documents/nr-reporting-summary-flat.pdf

Life sciences study design

All studies must disclose on these points even when the disclosure is negative.

Sample size	Sample sizes for all experiments are indicated in the figure legends and all the n's represent biological replicates unless otherwise specified. Sample sizes were determined based on experimental and budget constraints.
Data exclusions	No data were excluded from the analyses.
Replication	All attempts at replication were successful and are described in the text.
Randomization	To generate cell clones, single cells were randomly sorted into 96-well plates, and positive clones were then randomly selected for downstream analysis.
Blinding	Blinding was not relevant to this study because all experimental outcomes were quantitative and objectively measured.

Reporting for specific materials, systems and methods

We require information from authors about some types of materials, experimental systems and methods used in many studies. Here, indicate whether each material, system or method listed is relevant to your study. If you are not sure if a list item applies to your research, read the appropriate section before selecting a response.

Materials & experimental systems

n/a	Involved in the study	
☐	☒	Antibodies
☐	☒	Eukaryotic cell lines
☒	☐	Palaeontology and archaeology
☒	☐	Animals and other organisms
☒	☐	Clinical data
☒	☐	Dual use research of concern
☒	☐	Plants

Methods

n/a	Involved in the study	
☒	☐	ChIP-seq
☐	☒	Flow cytometry
☒	☐	MRI-based neuroimaging

Antibodies

Antibodies used	For ChIP-qPCR, we used the anti-IgG (Antibodies-Online, ABIN101961), and the anti-SIN3A (Novus Biologicals, NB600-1263) antibody for chromatin immunoprecipitation. Each sample was incubated with either anti-IgG (2.5 μg, Antibodies-Online, ABIN101961) or anti-SIN3A (2.5 μg, Novus Biologicals, NB600-1263).
Validation	The anti-SIN3A antibody was verified by western blotting on the manufacturer's website.<br>The anti-IgG antibody was verified by CUT&RUN on the manufacturer's website.

Eukaryotic cell lines

Policy information about cell lines and Sex and Gender in Research

Cell line source(s)	The WTC11 i3N iPSC line is a wild-type male cell line (PMID: 28966121) and was a gift from Li Gan's lab.<br>The human embryonic kidney (HEK293T) cell line is commonly used for virus production.
Authentication	Human iPSCs and differentiated excitatory neurons were authenticated using established stem cell and neuronal markers, respectively.
Mycoplasma contamination	All cells used in the present study were verified as mycoplasma contamination free.
Commonly misidentified lines<br>(See ICLAC register)	None of the cell lines used are commonly misidentified lines.

Plants

Seed stocks	N/A
Novel plant genotypes	N/A
Authentication	N/A

Flow Cytometry

Plots

Confirm that:

☒ The axis labels state the marker and fluorochrome used (e.g. CD4-FITC).

☒ The axis scales are clearly visible. Include numbers along axes only for bottom left plot of group (a 'group' is an analysis of identical markers).

☒ All plots are contour plots with outliers or pseudocolor plots.

☒ A numerical value for number of cells or percentage (with statistics) is provided.

Methodology

Sample preparation	The cells for flow cytometry analysis and fluorescence-activated cell sorting (FACS) were dissociated into single cells using Accutase (STEMCELL Technologies, 07920) for iPSCs and Papain (Sigma, P4762) for excitatory neurons. The iPSCs were resuspended with FACS buffer (1× DPBS, 2mM EDTA, 25mM HEPES pH7.0, and 1% FBS), and neurons were resuspended with HBSS buffer (Gibco, 14175095) with 0.5% FBS.
Instrument	Flow cytometry analyses were performed on BD LSR II and BD LSRFortessa Flow Cytometers. FACS experiments were conducted on a BD FACSAria II instrument using a 100-μm nozzle.
Software	FACSDiva<br>FlowJo (v10.7.2)
Cell population abundance	For generation of reporter iPSC lines, single cells were sorted into 96-well plates, and positive clones were validated by flow cytometry, confirming uniform reporter expression. For CREST-seq screens, sorted cells were re-analyzed on the cell sorter, with >98% of cells falling within the original sorting gate.
Gating strategy	We used the same gate setting for both flow cytometry analysis and FACS. First, cells were separated from debris based on forward scatter area (FSC-A) and side scatter area (SSC-A). Then, single cells were separated using single cell gates based on

the area and width metrics of forward scatter (FSC-A vs. FSC-W) and side scatter (SSC-A vs. SSC-W). Further, the gates for EGFP and mCherry signal baselines were set using cells without EGFP and mCherry signals.

☒ Tick this box to confirm that a figure exemplifying the gating strategy is provided in the Supplementary Information.
